# Progress in State Medicine

**Published:** 1899-01-14

**Authors:** 


### Progress in State Medicine.

Water.—Delépine[1] makes an entirely new departure in the method of establishing a standard for the interpretation of the results of bacteriological examination of water. The standard of impurity has been up to the present exceedingly variable, but generally 500 colonies of bacteria per cubic centimetre of water has been regarded as the maximum limit consistent with purity, and by all a water containing 1,000 colonies per cubic centimetre has been condemned as unfit for use. Delépine adopts a comparative method, taking as his standard the water from a feeder of the supply which he has by personal investigation proved to be free from sources of contamination. Bacteriological examination is made of the water of this feeder during both fine and wet weather, and careful note is made of the ground slope, of paths, of the presence of cattle, and the relation of traffic to this feeder by which all others in connection with the particular supply are to be tested. Rain, it was found, increases the pollution about 300 per cent. In collecting the samples care must be taken not to disturb the banks or bed of the streamlet. To prevent the entrance of sediment, which is always stirred up if an ordinary bottle is plunged into the stream, special sterilised vacuum flasks are employed. These have long glass necks drawn out to a point. The neck is immersed in the water, the point broken off with sterile forceps, the water then rising in the flask. When withdrawn, the flask is closed by fusing the glass point. Before taking specimens from hydrants the water should be allowed to run for twenty minutes. It is then received in self-closing sterilised tins, and from these into the sterilised vacuum tubes, For cultures, made for the purpose of estimating the presence or absence of pollution, gelatine plates incubated at 22 deg. C., and of standard alkalinity, best suffice to demonstrate both pathogenic and saprophytic organisms. The writer makes the noteworthy observation that if there be excess of organic impurity in a water, it will be found that if it is kept for 18 hours at 37 deg. C., and then plated, the bacilli have undergone marked multiplication, whilst in a pure water similarly treated a diminution results. As subsidiary methods of bacteriological examination, growth in alkaline broth and the inoculation of animals are mentioned.

Samples taken in the manner described above need not be plated at once, but if packed in ice an interval of several hours will not interfere with the result. Every plate should be kept at 22 deg. C., and the number of colonies counted on the fourth or fifth day and the varieties at the end of a week. Possible sources of error are due to keeping the water too long at the room temperature before plating, measuring by bulk rather than by weight, counting colonies on different days, and only counting these on a portion of the plate. Special cases are cited. One uncontaminated feeder showed 70-80 colonies per cubic centimetre in fine, and 210-280 in wet weather. This is a good average, 100 per cubic centimetre in fine weather being excessive. A warning is entered against relying upon filtration as a safeguard against pollution. Protection of the collecting area is the great protection, although in times of storm, filtration is essential. Each service reservoir should have a separate filter.

The necessity for a uniform method of studying the bacterial flora of water has already been recognised in America, and a committee of the American Public Health Association[2] has drawn up a scheme which is to act as a basis of a common method of studying, describing, and differentiating water bacteria. The report is accompanied by a series of charts illustrating morphological and biological data which should prove invaluable in the task of isolating and recognising varieties of organisms. Undoubtedly the most valuable part of the work is the information concerning the means of obtaining standard media, and thus permitting greater uniformity of description and identification. Especial attention is paid to (*a*) cultural characteristics, (*b*) biochemical features, (*c*) pathogenesis. The adoption of some such scheme would be of great service to this country. Similarly, in the case of river waters, it is of the utmost importance that some uniform method should be employed with regard to the times and seasons at which samples should be taken for both chemical and biological examination. Kibrehl,[3] from bacteriological examinations of the Moldan before and after its entrance to the City of Prague, finds that the number of bacterial colonies increases with rising and decreases with falling water. This change is due to the foul influx following heavy rains (street sweepings, &c.); to the increased rapidity of flow, and consequent delay in sedimentation; and to the altered conditions of light. The water is best sampled when, with a falling stream, the river approaches its normal depth. If taken at high or low water, or with a rising stream, gross errors may arise. With much organic pollution the number of organisms is largely dependent on the temperature. The pollution of river water by chemical waste may lead to the death of fish, although by chemical examination the stream may not prove abnormally foul. Thörner,[4] investigating a case of this kind, attributes the deleterious effect to the diminution of the quantity of oxygen present. In two cases quoted analysis showed oxygen to be entirely absent in the samples, having been used in converting ferrous salts of the waste into ferric hydrate. This lack of aëration could, of course, only take place in inland waters. At the present time our largest towns are seeking to obtain their water supplies from distant catchment areas, so as to secure a maximum supply with the minimum of pollution. Liverpool, Glasgow, and Manchester are already thus provided, and the supply for Birmingham, drawn from storage reservoirs in the Welsh hills, 73 miles away, is now in course of construction. That such storage reservoirs and sedimentation tanks may themselves become polluted has been demonstrated by recent events at Maidstone and Lynn, and it therefore behoves the householder still further to protect himself by providing an efficient filter for all water to be used for drinking purposes. Non-pressure filters, as described in the report of Cartwright Wood and Sims Woodhead, have already been dealt with in these columns. The second part of their inquiry[5] has reference to pressure filters. Of these twelve varieties were tested, and six found to give satisfactory results with regard to their powers of conferring protection against the communication of water-borne disease and yielding a sufficient supply for domestic purposes. These filters are the Pasteur-Chamberland, Berkefeld, Porcelaine D'Amiante, Pukall, Slack and Brownlow's, and Duff's patent germ-proof filter. The only efficient media for filters are porcelain, compressed siliceous earth, and natural stone. The action of these filters depends on the fineness of their pores, and intimate contact with the walls of the pores appears to have a devitalising effect upon water organisms. The thickness of the filtering material is not a factor of importance in comparison with the size of the pores. Water, even when highly polluted with sewage is not a suitable medium for disease germs, and such germs as those of cholera or enteric fever do not find a suitable breeding ground in any of the ordinary filter media.

Fine porcelains, although giving excellent bacteriological results, yield a scanty supply of water. A larger output is realised by the diatomaceous earth filters (Berkfeldt), and natural stone such as used in Duff's patent filter affords an abundant supply. The rapidity and thoroughness with which the filtering material can be cleaned is an important factor. In this respect porcelain is the most difficult, whilst with Berkfeldt material the risk of damaging the brittle substance is the greatest. Pressure filters attached to taps should yield sufficient water to supply all domestic requirements. The filter may with advantage be fixed in the cistern where it cannot be tampered with. The present army field service filters are inefficient.

[1] "Bacteriological Survey of Surface-Water Supplies," Journal of State Medicine Ap.—Aug., 1898. [2] Brit. Med. Jour., July 2, 1898. [3] Archiv. für Hygiene, xxx., 32. [4] Forschungsbericht für Lebensmittel, 1897, 172. [5] Brit. Med. Jour., Jan. 22, 1898.

